# Ddx3xa mutations drive cardiac defects in a zebrafish model via dysregulation of wnt/β-catenin signaling

**DOI:** 10.3389/fmolb.2025.1689202

**Published:** 2025-11-27

**Authors:** Yu Chen, Mei Lin, Ping Zhu, Haochen Wang, Zhongbei Jiao, Kexing Yi, Xueting Yang, Yingshuo Zhang, Xiaoyan Cai, Wuzhou Yuan, Yongqing Li, Zhigang Jiang, Yuequn Wang, Fang Li, Xiushan Wu, Xiongwei Fan

**Affiliations:** 1 The Center for Heart Development, College of Life Science, Hunan Normal University, Changsha, China; 2 Guangdong Provincial Key Laboratory of Pathogenesis, Targeted Prevention and Treatment of Heart Disease, Guangzhou Key Laboratory of Cardiac Pathogenesis and Prevention, Guangzhou, China; 3 Medical Research Center, Guangdong Provincial People’s Hospital (Guangdong Academy of Medical Sciences), Southern Medical University, Guangzhou, China

**Keywords:** *ddx3xa*, zebrafish, CRISPR/Cas9, cardiac defects, wnt/β-catenin signaling, IWR-1 rescue

## Abstract

**Introduction:**

Mutations in the DDX3X gene are the primary cause of DDX3X syndrome, with over 800 diagnosed families worldwide. DDX3X is also recognized as a single-gene driver for rare syndromes associated with epilepsy, autism, and developmental disorders. Clinical studies suggest potential links between DDX3X mutations and various cardiac comorbidities. However, there is no report on whether Ddx3xa knockout leads to cardiac phenotypes or whether a zebrafish *ddx3xa* gene knockout model has been used for such research. This study is based on the high genomic conservation between zebrafish and humans, utilizing a zebrafish model to investigate the potential links between DDX3X mutations and various cardiac comorbidities, as well as the underlying mechanisms.

**Methods:**

A *ddx3xa* knockout model was constructed using CRISPR/Cas9 technology. To elucidate the molecular mechanisms, we performed transcriptome-wide profiling via RNA-Seq to identify differentially expressed genes and dysregulated signaling pathways. The spatiotemporal expression patterns of key genes were assessed using whole-mount in situ hybridization (WISH). Additionally, the critical role of Wnt/β-catenin signaling in the mutant phenotype was further validated using the Wnt inhibitor IWR-1.

**Results:**

Homozygous knockout (*ddx3xa* ^*−/−*^) embryos exhibited developmental delay, trunk malformations, and severe cardiac abnormalities, including pericardial edema, defective cardiac looping, and cardiac contractile dysfunction. Ribonucleic Acid Sequencing (RNA-seq) analysis of *ddx3xa* ^−/−^ zebrafish at 72 h post-fertilization (hpf) revealed significant enrichment in pathways related to actin cytoskeleton organization, calcium signaling, cardiac and vascular smooth muscle contraction, and Wnt signaling. Quantitative Real-Time Reverse Transcription Polymerase Chain Reaction (QRT-PCR) and *in situ* hybridization confirmed dysregulated expression of key cardiac development genes (*bmp4*, *actn2b*, *tbx5*, *nppb*) and significantly impaired cardiac function. Given the role of Wnt signaling in cardiogenesis, we further analyzed this pathway and found that *ddx3xa* knockout upregulated the key Wnt/β-catenin transcription factor Tcf/Lef1 (T Cell Factor/Lymphoid Enhancer Factor 1) and disrupted its target genes (*bmp4*, *tbx5*) expression. Crucially, treatment of 72 hpf mutant embryos with the Wnt inhibitor IWR-1 partially rescued both the cardiac malformations and the aberrant expression of its target genes.

**Discussion:**

This study provides the first evidence that *ddx3xa* regulates cardiac morphogenesis by modulating the Wnt/β-catenin signaling pathway, offering direct experimental insight into the mechanisms underlying cardiac comorbidities in DDX3X syndrome. It also highlights the unique value of the zebrafish model for dissecting conserved pathogenic pathways and exploring targeted therapeutic strategies.

## 1 Introduction

Proteins belonging to the DDX family are characterized by a highly conserved structural motif, the Asp-Glu-Ala-Asp (D-E-A-D) amino acid sequence (Linder and Jankowsky, 2011; Mo et al., 2021). DEAD-box proteins possess essential functions, including ATP hydrolysis, modulation of helicase activity, and participation in RNA metabolism as RNA-binding proteins (Jankowsky et al., 2011). Research indicates that DEAD-box family proteins play regulatory roles in critical cellular processes such as the cell cycle, apoptosis, and tumorigenesis, with clinical studies linking DDX protein mutations to the pathogenesis of various diseases, including cancer (Mo et al., 2021). DDX3 is a member of the DEAD-box helicase family. The human genome encodes two paralogous genes: *DDX3X* and its homolog *DDX3Y* (Kim et al., 2001). The *DDX3* gene encodes a highly conserved RNA helicase that is crucial for RNA metabolism, translation initiation, and innate immune responses, and it has emerged as a significant therapeutic target in oncology (Bol et al., 2015; Devasahayam Arokia Balaya et al., 2025). Specifically, mutations in the *DDX3X* gene represent the primary cause of DDX3X syndrome, a neurodevelopmental disorder diagnosed in over 800 families worldwide. This syndrome accounts for approximately 3% of intellectual disability (ID) cases and exhibits a strong female predominance (Tang et al., 2021; von Mueffling et al., 2024). Studies have established clear associations between *DDX3X* mutations and a spectrum of neurological conditions, including epilepsy, autism spectrum disorder (ASD), and intellectual disability, alongside links to immune dysregulation and oncogenesis (Boitnott et al., 2021; von Mueffling et al., 2024). Although clinical observations have implicated *DDX3X* mutations in cardiac comorbidities (Dikow et al., 2017), the molecular mechanisms underpinning these cardiac contractile impairments remain largely unexplored.

The *DDX3X* gene is highly conserved across eukaryotes. It exhibits 98.6% amino acid identity between humans and mice, and targeted knockout of the murine *Ddx3x* gene results in early embryonic lethality, underscoring its critical role in early embryonic survival (Owsianka and Patel, 1999; Ditton et al., 2004; Padmanabhan et al., 2016). In hepatocytes, DDX3X protects against drug-induced acute liver injury by modulating stress granule formation and oxidative stress responses (Luo et al., 2023). Pathogenic *DDX3X* mutations impair RNA metabolism and neurogenesis during fetal cortical development (Lennox et al., 2020). Clinical data from breast cancer patients indicate that high expression of the RNA helicase DDX3 is significantly associated with shortened overall survival and bone metastasis; consequently, small-molecule inhibitors targeting DDX3X are being explored as therapeutic agents for breast cancer bone metastasis (Winnard et al., 2024). Furthermore, DDX3X establishes a dynamic balance between viral propagation and host defense within the antiviral innate immune response (Ma et al., 2025). While the roles of DDX3X in liver injury, neurodevelopment, cancer, and innate immunity have been partially elucidated, its association with cardiac pathologies remains largely unexplored. Thus, elucidating the regulatory mechanisms of DDX3X within the cardiac system necessitates *in vivo* investigations using model organisms.

The zebrafish (*Danio rerio*) has emerged as a powerful model for studying developmental disorders due to its high degree of genetic and physiological conservation with humans (Dooley and Zon, 2000). Approximately 84% of human disease genes possess orthologs in zebrafish, and its transparent embryos facilitate real-time observation of organogenesis, making it particularly valuable for cardiovascular research (Lawrence, 2007; Dahme et al., 2009). The zebrafish genome harbors two paralogous *ddx3x* genes, *ddx3xa* and *ddx3xb*, both exhibiting high conservation with human DDX3X (Duan et al., 2024). Given their highly similar sequences and overlapping expression patterns, *ddx3xa* and *ddx3xb* display functional redundancy in certain contexts; for instance, the combined deficiency of DDX3X function leads to defects in neurogenesis, a process where these paralogs compensate for each other (Duan et al., 2024). However, studies targeting *ddx3xb* function specifically have revealed that the Ddx3xb helicase regulates the maternal-to-zygotic transition (MZT) in zebrafish embryogenesis through liquid-liquid phase separation (Shi et al., 2022). Thus, existing evidence indicates that while *ddx3xa* and *ddx3xb* share functional redundancy, they also possess distinct regulatory mechanisms. Previous research has demonstrated that loss of *ddx3xa* in zebrafish results in developmental delay and brain abnormalities (Duan et al., 2024). Nevertheless, its specific role in cardiac contractile function remains undefined and constitutes a primary focus of this investigation.

Numerous studies have established that Wnt signaling pathways are activated and play pivotal regulatory roles in both physiological and pathological processes, including cardiovascular diseases and cancer (Nusse and Clevers, 2017). Among these, the Wnt/β-catenin signaling pathway is evolutionarily highly conserved. It participates extensively in nearly all stages of cardiac development, encompassing early specification, cardiomyocyte differentiation, heart tube looping, chamber and valve formation, and conduction system patterning (Naito et al., 2006; Li et al., 2022). Notably, over half of patients diagnosed with arrhythmogenic right ventricular cardiomyopathy (ARVC) harbor mutations in genes encoding desmosomal proteins (Towbin, 2011). These mutant desmosomal proteins competitively bind to the transcription factors Tcf/Lef, displacing β-catenin. This disrupts canonical Wnt signaling and promotes the transdifferentiation of cardiomyocytes into adipocytes and fibroblasts, leading to adipogenic replacement and the development of arrhythmogenic cardiomyopathy (Garcia-Gras et al., 2006; Lombardi et al., 2011). Key downstream target genes of the canonical Wnt pathway include *C-Myc*, *cyclin D1*, *C-fos*, *BMP4*, *TBX5*, *mesp1*, and *actn2b*, among others (Nishimoto et al., 2015; Medina et al., 2016; Ye et al., 2019; Zhang et al., 2024). Intriguingly, studies have identified an interaction between zebrafish *ddx3x* and *csnk2a1* (casein kinase 2 alpha 1), a known component of the Wnt signaling machinery, suggesting a potential regulatory role for *ddx3x* in Wnt/β-catenin signaling (Wang et al., 2023). However, it remains unclear whether *ddx3x* specifically modulates Wnt signaling during cardiac morphogenesis. Furthermore, there is currently no evidence regarding whether *ddx3x* knockout models induce cardiac phenotypes, nor have zebrafish *ddx3x* gene knockout models been established.

In this study, we employed CRISPR/Cas9 technology to generate a *ddx3xa* knockout zebrafish model (Sun et al., 2020), specifically to investigate its role in cardiac development and function. We observed that loss of *ddx3xa* function resulted in embryonic developmental abnormalities, including developmental delay, pericardial edema, bradycardia, and defective cardiac looping. qRT-PCR and *in situ* hybridization analyses demonstrated that *ddx3xa* knockout led to the upregulation of key Wnt/β-catenin pathway components, such as the transcription factor Tcf/Lef1. This dysregulation consequently disrupted the expression of critical downstream cardiac genes (*bmp4*, *actn2b*, *tbx5*, *nppb*), ultimately leading to severely impaired cardiac defects. Crucially, pharmacological inhibition of the Wnt/β-catenin pathway using the small-molecule inhibitor IWR-1 partially rescued both the aberrant expression of these molecular markers and the associated cardiac defects. These findings underscore a central role for *ddx3xa* in mediating cardiac regulation through the Wnt/β-catenin signaling pathway, demonstrating that *ddx3xa* governs zebrafish embryonic cardiac defects by modulating Wnt signaling activity. Collectively, our work establishes the essential role of *ddx3xa* in cardiac morphogenesis and provides crucial mechanistic insights into the pathogenesis of cardiac comorbidities associated with DDX3X syndrome, offering a valuable potential disease model.

## 2 Results

### 2.1 Homology and evolutionary analysis of the zebrafish *ddx3xa* gene structure

**FIGURE 1:**
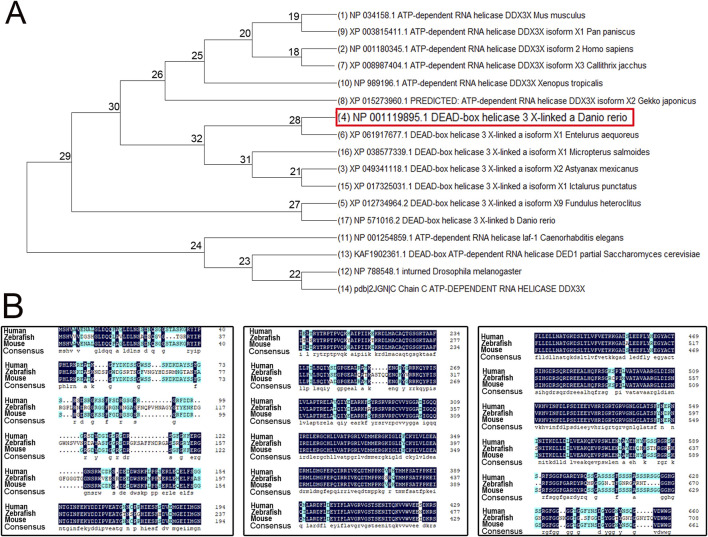
Phylogenetic tree and amino acid sequence analysis of DDX3X across species. **(A)** Phylogenetic tree of DDX3X homologs from diverse species. **(B)** Amino acid sequence alignment of *Homo sapiens* DDX3X, *Mus musculus* DDX3X, and *Danio rerio* Ddx3xa.

To investigate the evolutionary conservation and functional significance of *ddx3xa*, we performed phylogenetic and sequence homology analyses. A phylogenetic tree was constructed using MEGA X software based on amino acid sequences of DDX3X homologous proteins from multiple species (*Escherichia coli*, *Saccharomyces cerevisiae*, *Drosophila melanogaster*, *Caenorhabditis elegans*, *Xenopus tropicalis*, *Pan paniscus*, *Homo sapiens*, *Mus musculus*, and various teleost fishes). The results revealed close evolutionary relationships with minimal genetic divergence among these species (Figure 1A), suggesting high conservation of DDX3X structure and function throughout evolution. Further sequence alignment using DNAMAN software demonstrated >75% amino acid sequence homology between human DDX3X, mouse DDX3X, and zebrafish Ddx3xa. Notably, the RNA-binding domain exhibited exceptional conservation (Figure 1B). These findings provide further confirmation of Ddx3xa’s evolutionary conservation and support the rationale for utilizing zebrafish as a model to study its biological functions.

### 2.2 Expression pattern of *ddx3xa* in early zebrafish embryos revealed by whole-mount *in situ* hybridization and qRT-PCR

To delineate the spatial and temporal expression profile of *ddx3xa* during early development, whole-mount *in situ* hybridization (WISH) was performed on zebrafish embryos at six developmental stages: 0.2, 12, 24, 36, 48, and 72 h post-fertilization (hpf), using a synthesized *ddx3xa*-specific probe.

**FIGURE 2:**
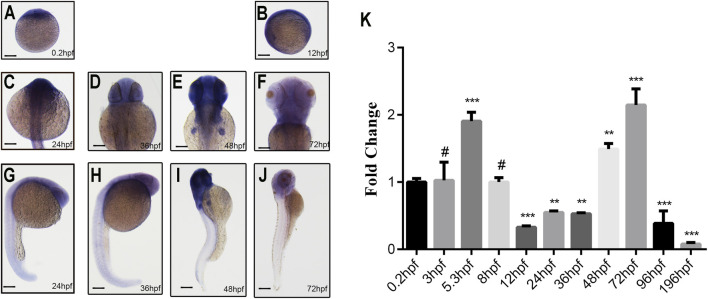
Spatiotemporal expression pattern of *ddx3xa* by whole-mount *in situ* hybridization and qRT-PCR analysis. **(A–J)** Spatiotemporal expression of *ddx3xa* at various developmental stages visualized by whole-mount *in situ* hybridization. Scale bar: 250 μm (applies to A–J). **(A)** 0.2 hpf; **(B)** 12 hpf; **(C)** 24 hpf; **(D)** 36 hpf; **(E)** 48 hpf; **(F)** 72 hpf; **(G)** Dorsal view, 24 hpf; **(H)** Dorsal view, 36 hpf; **(I)** Dorsal view, 48 hpf; **(J)** Dorsal view, 72 hpf. **(K)** Quantitative real-time PCR (qRT-PCR) analysis of relative *ddx3xa* transcript levels at various developmental time points (0.2, 3, 5.3, 8, 12, 24, 36, 48, 72, 96, 196 hpf). Expression levels were normalized to the 0.2 hpf time point. (n = 3; **P < 0.01, ***P < 0.001).

WISH results revealed ubiquitous *ddx3xa* expression at 0.2 hpf and 12 hpf (Figures 2A,B). At 24 hpf and 36 hpf, expression remained widespread throughout the embryo, with strong signals detected in the brain, skeletal muscles, and tail, exhibiting the highest intensity in the brain (Figures 2C,D,G,H). By 48 hpf, expression became more restricted, primarily localizing to the brain and pectoral fin buds, with diminished expression in the tail compared to earlier stages (Figures 2E,I). At 72 hpf, expression was confined specifically to the brain, heart, and pectoral fin buds (Figures 2F,J). These findings suggest that *ddx3xa* participates in various stages of zebrafish early embryogenesis, with predominant roles in brain and heart development.

To quantitatively assess *ddx3xa* transcript levels across development, qRT-PCR analysis was conducted on embryos collected at 11 time points: 0.2, 3, 5.3, 8, 12, 24, 36, 48, 72, 96, and 196 hpf. *Ddx3xa* transcripts were detectable as early as 0.2 hpf and persisted throughout early embryonic development. Notably, transcript levels peaked at 72 hpf (Figure 2K). This expression profile indicates that *ddx3xa* likely functions as a widely expressed, maternally derived gene regulating early zebrafish embryogenesis, with its expression sustained across the entirety of early embryonic development.

### 2.3 Construction of the *ddx3xa*-Knockout zebrafish line

**FIGURE 3:**
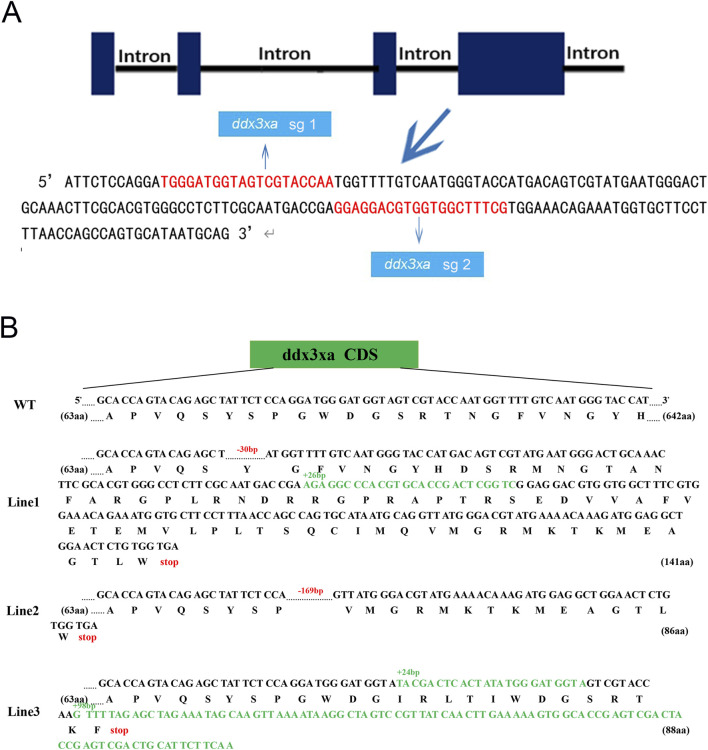
Schematic representation of *ddx3xa* knockout in zebrafish. **(A)** Design of sgRNAs targeting *ddx3xa*. Horizontal lines represent *ddx3xa* genomic DNA, blue rectangles denote exons, and red text indicates target site sequences. **(B)** Schematic of three heritable *ddx3xa* mutant alleles generated by CRISPR/Cas9 and their resultant protein sequences.

To investigate the role of *ddx3xa* in early zebrafish development, we established a CRISPR/Cas9-mediated knockout line using two single-guide RNAs (sgRNAs) targeting exon 4 of *ddx3xa*: Target 1 (5′-TGGGATGGTAGTCGTACCAA-3′) and Target 2 (5′-GGAGGACGTGGTGGCTTTCG-3′) (Figure 3A). This approach generated three stable lines (Lines 1–3) carrying frameshift mutant alleles. Sanger sequencing confirmed line-specific mutations: Line 1 exhibited a 102 bp intronic deletion with a 30 bp exonic deletion and 26 bp insertion; Line 2 showed a 169 bp exonic deletion plus 82 bp intronic deletion; and Line 3 contained a 24 bp insertion with 98-bp exonic insertion (Figure 3B). Preliminary morphological and genotypic analyses confirmed that all three mutant lines (Lines 1–3) exhibited indistinguishable core phenotypes. To ensure experimental consistency and reproducibility, all subsequent experiments were uniformly performed using Line 2.

**FIGURE 4:**
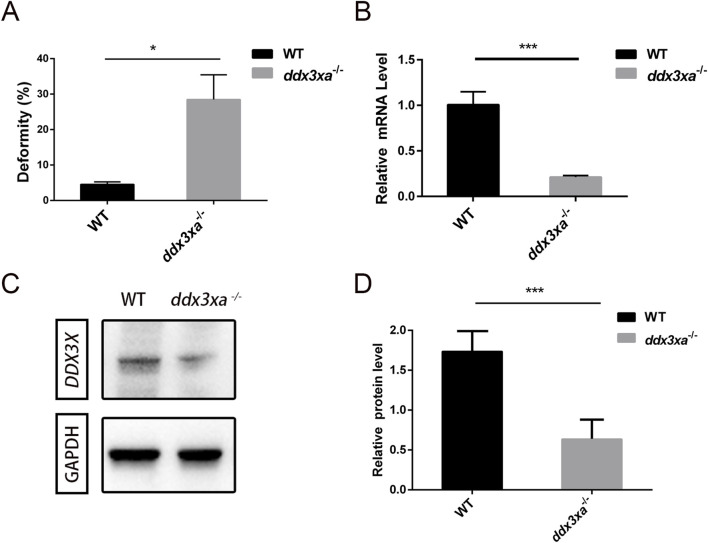
Malformation rate quantification and validation of *ddx3xa* knockout efficiency in zebrafish. **(A)** Malformation rate in *ddx3xa* ^−/−^ zebrafish at 48 hpf (**P < 0.01 vs. wild-type). **(B)** qRT-PCR analysis of *ddx3xa* mRNA levels in 72-hpf embryos showing significant downregulation in mutants (n = 3; *P < 0.05, ***P < 0.001). **(C)** Representative Western blot of Ddx3xa protein expression in 72-hpf larvae. Gapdh served as a loading control. **(D)** Quantitative densitometric analysis of Ddx3xa protein levels in wild-type and *ddx3xa* ^−/−^ larvae at 72 hpf.

Stereomicroscopic observation revealed developmental delays in mutants initiating at 2 hpf and persisting through 24 hpf (Supplementary Figure S1A). By 48 hpf, *ddx3xa* ^−/−^ embryos displayed severe morphological abnormalities. Quantitative analysis demonstrated a 25.26% malformation rate in mutants (Figure 4A; Supplementary Figure S1B) - representing a nearly 6-fold increase compared to wild-type (WT) controls (4.4%) - indicating profound disruption of early development.

Knockout efficiency was validated by qRT-PCR at 72 hpf, showing significantly reduced *ddx3xa* mRNA levels in mutants versus WT (Figure 4B). Western blot analysis further confirmed markedly decreased Ddx3xa protein expression in *ddx3xa* ^−/−^ embryos (Figures 4C,D), consistent with premature translational termination due to frameshift mutations. These results collectively confirm successful establishment of the *ddx3xa*-knockout line.

### 2.4 *ddx3xa* knockout induces cardiac malformations and functional deficiencies in zebrafish embryos

**FIGURE 5:**
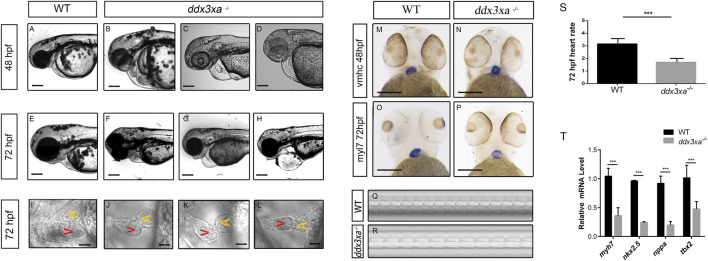
*ddx3xa* knockout induces early cardiac malformation and dysfunction in zebrafish. **(A–H)** Phenotypic analysis of zebrafish hearts. **(A)** Normal cardiac phenotype in wild-type (WT) zebrafish at 48 hpf. **(B–D)** Cardiac phenotypes in *ddx3xa* ^−/−^ zebrafish at 48 hpf. **(E)** Normal cardiac phenotype in WT zebrafish at 72 hpf. **(F–H)** Cardiac phenotypes in *ddx3xa* ^−/−^ zebrafish at 72 hpf. Scale bar: 500 µm (applies to A–H). **(I)** Dorsal view of the heart in WT zebrafish at 72 hpf. **(J–L)** Dorsal views of hearts in *ddx3xa* ^−/−^ zebrafish at 72 hpf. Scale bar: 25 µm (applies to I–L). **(M–N)** Whole-mount *in situ* hybridization (WISH) with vmhc probe at 48 hpf. *ddx3xa* ^−/−^ larvae display irregular ventricular contours compared to WT (scale bar: 250 μm). **(M)** *vmhc* WISH in WT zebrafish at 48 hpf. **(N)** vmhc WISH in *ddx3xa* ^−/−^ zebrafish at 48 hpf. **(O–P)** *myl7* probe WISH at 72 hpf. *ddx3xa* ^−/−^ larvae exhibit defective atrioventricular looping and atrial hypoplasia compared to WT (scale bar: 250 μm). **(O)** *myl7* WISH in WT zebrafish at 72 hpf. **(P)** *myl7* WISH in *ddx3xa* ^−/−^ zebrafish at 72 hpf. **(Q–T)** Heartbeat analysis in WT and *ddx3xa* ^−/−^ zebrafish at 72 hpf. **(Q)** Representative heartbeat trace of WT zebrafish at 72 hpf. **(R)** Representative heartbeat trace of *ddx3xa* ^−/−^ zebrafish at 72 hpf. **(S)** Statistical analysis of heart rate (n = 3; ***P < 0.001). **(T)** qRT-PCR analysis of *myh7*, *nkx2.5*, *nppa*, and *tbx2* transcript levels in WT and *ddx3xa* ^−/−^ larvae at 72 hpf (n = 3; **P < 0.01, ***P < 0.001).

Morphological analysis of the heart revealed that a subset of mutant embryos developed pericardial edema, cardiac linearization, and impaired looping from 48 hpf. These defects were consistently observed and persisted through at least 72 hpf, in contrast to their wild-type counterparts (Figures 5A–H). Quantitative analysis of these cardiac malformations at 48 hpf revealed a significant increase in the mutant group, as measured by the percentage of embryos with pericardial edema, cardiac linearization, and impaired looping process (Supplementary Figures S2,S3; Supplementary Table S1).

To better assess the cardiac morphological changes induced by Ddx3xa knockout, 72 hpf zebrafish larvae were immobilized in a high concentration of methylcellulose and positioned dorsally on a slide for observation. This revealed that wild-type (WT) larvae at 72 hpf displayed an atrioventricular canal and expanded heart chambers arranged side-by-side. In contrast, *ddx3xa* ^*−/−*^ larvae exhibited failure of cardiac looping, presenting a linear heart tube (Figures 5I–L).

To elucidate the molecular basis of the cardiac developmental defects, we performed spatial expression pattern analysis using whole-mount *in situ* hybridization (WISH) with cardiac-specific markers. At 48 hpf, staining with the ventricular marker *vmhc* (ventricular myosin heavy chain) showed a significantly irregular ventricular margin in mutant larvae (Figures 5M,N), suggesting impaired ventricular specification or morphogenesis. By 72 hpf, staining with the pan-cardiac marker *myl7* (myosin light chain 7) revealed more profound defects: mutant hearts displayed not only abnormal atrioventricular looping but also pronounced atrial hypoplasia (Figures 5O,P). This indicates that loss of *ddx3xa* disrupts the normal development and patterning of both the atrium and ventricle.

To further assess whether cardiac function was compromised in the zebrafish larvae, cardiac contraction rates were measured over a 10-s interval in both WT and *ddx3xa* ^−/−^ larvae at 72 hpf and statistically analyzed. While rhythmic, the heart rate of *ddx3xa* ^−/−^ zebrafish was significantly slower than that of WT larvae.

To identify the molecular drivers underlying these morphological phenotypes, we performed qRT-PCR analysis on 72 hpf larvae to quantify the expression levels of key cardiac developmental regulatory genes. The results demonstrated significant downregulation of *myh7* (myosin heavy chain 7), *nkx2.5*, *nppa*, and *tbx2* in mutants (Figure 5T). These genes are involved in critical biological processes including cardiomyocyte differentiation, cardiac progenitor maintenance, morphogenesis, and contractile function regulation. Their coordinated downregulation provides a direct molecular explanation for the observed severe cardiac malformations (such as looping failure and atrial hypoplasia) and functional impairment.

In summary, this study demonstrates that *ddx3xa* knockout induces severe cardiac malformation and dysfunction in zebrafish embryos by disrupting the cardiac looping process, impairing cardiomyocyte differentiation, and causing abnormal heart rate. This establishes an important *in vivo* model for gaining deeper insights into the pathogenesis of cardiac comorbidities associated with DDX3X syndrome.

### 2.5 Transcriptome sequencing analysis reveals abnormal regulation of wnt signaling pathway and cardiac development-related pathways in *ddx3xa*-deficient zebrafish

#### 2.5.1 Global transcriptome analysis

**FIGURE 6:**
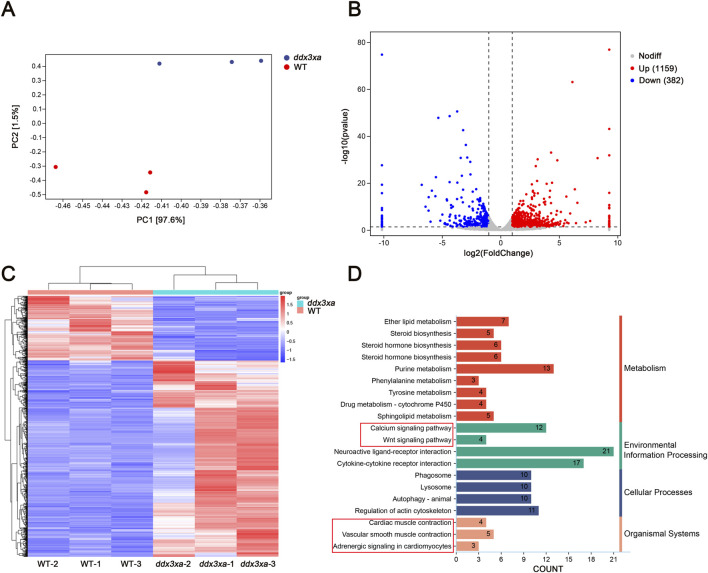
Transcriptomic alterations in *ddx3xa* ^−/−^ zebrafish at 72 hpf. **(A)** Principal component analysis (PCA) of the transcriptome revealed clear separation between WT and *ddx3xa* ^−/−^ groups along PC1, which accounted for 97.6% of the total variance (PC2: 1.5%). Sample distribution: wild-type (WT) controls (red) versus *ddx3xa* ^−/−^ mutants (blue). **(B)** Volcano plot of differentially expressed genes (DEGs): downregulated (blue), nonsignificant (gray), upregulated (red). **(C)** Hierarchical clustering heatmap: rows represent genes, columns represent samples. Expression levels scaled from high (red) to low (blue). **(D)** KEGG pathway enrichment analysis.

Principal component analysis (PCA) revealed clear separation between WT and *ddx3xa* ^*−/−*^ groups along the first principal component (PC1), which accounted for 97.6% of the total transcriptional variance (PC2: 1.5%). The distinct clustering of biological replicates within each group indicated high reproducibility, while the pronounced separation along PC1 demonstrated that the *ddx3x* deletion was the dominant source of transcriptional variation (Figure 6A; Supplementary Table S2). RNA-Seq analysis of wild-type (WT) and *ddx3xa* ^−/−^ zebrafish at 72 hpf identified 1,541 differentially expressed genes (DEGs), comprising 1,159 upregulated and 382 downregulated genes in mutants relative to WT controls (Figure 6B). Hierarchical clustering analysis of all DEGs is presented in Figure 6C. Kyoto Encyclopedia of Genes and Genomes (KEGG) pathway enrichment revealed significant alterations in actin cytoskeleton organization, neuroactive ligand-receptor interactions, calcium signaling, cardiac and vascular smooth muscle contraction, and Wnt signaling pathway (Figure 6D). Thus, this global transcriptomic profiling establishes a foundational link between *ddx3xa* deficiency and cardiac pathogenesis. The identified DEGs provide a valuable resource for pinpointing key cardiac regulators. Crucially, the enriched pathways directly elucidate the observed phenotypes: actin cytoskeleton and calcium signaling anomalies underlie the contractile dysfunction and bradycardia, while the significant enrichment of the Wnt pathway, alongside others, suggests a synergistic multi-pathway disruption. This evidence positions Wnt signaling as a prime candidate for the core regulatory mechanism, guiding subsequent targeted investigation.

#### 2.5.2 qPCR validation of key pathway genes

**FIGURE 7:**
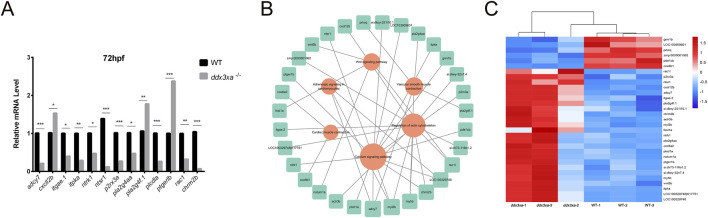
Validation and pathway analysis of cardiac development genes in *ddx3xa* ^−/−^ zebrafish. **(A)** qRT-PCR validation of genes related to cardiac contraction, vascular smooth muscle contraction, calcium signaling, and actin cytoskeleton regulation in *ddx3xa* ^−/−^ mutants versus wild-type (WT) at 72 hpf (n = 3; *P < 0.05, **P < 0.01, ***P < 0.001). **(B)** Pathway-gene interaction network for enriched cardiac development pathways generated using Cytoscape. **(C)** Heatmap of RNA-Seq expression profiles for enriched genes across key cardiac development pathways.

To validate RNA-Seq reliability, we performed qPCR analysis on candidate genes from enriched pathways: actin cytoskeleton regulation, calcium signaling, Wnt signaling, cardiac and vascular smooth muscle contraction, and cardiomyocyte adrenergic signaling. Consistent with transcriptomic data, *ddx3xa* ^−/−^ mutants showed significant downregulation of *adcy7*, *itgae.1*, *itpka*, *ntrk1*, *ntsr1*, *p2rx3a*, *pla2g4aa*, *plcd1a*, *rac1*, and *chrm2b* (P < 0.05), while *cxcl12b*, *pla2g4f.1*, and *ptger1b* were significantly upregulated (P < 0.05) (Figure 7A). Notably, aberrant expression of adrenergic signaling-related genes (*adcy7*, *ntsr1*) and myocardial contraction regulators (*plcd1a*, *rac1*) suggests *ddx3xa* deficiency disrupts cardiac development through impaired cardiomyocyte signaling and contractile function.

Using Cytoscape, we generated integrated pathway-gene networks for enriched cardiac development pathways (Figure 7B), with corresponding expression patterns visualized in an RNA-Seq-based heatmap (Figure 7C).

#### 2.5.3 *ddx3xa* knockout disrupts wnt signaling in zebrafish

Initial experiments revealed cardiac malformations in *ddx3xa* ^−/−^ zebrafish larvae, suggesting that gene knockout may perturb the expression of key cardiac developmental regulators. Supporting this, prior RNA-seq data and studies in *Xenopus tropicalis* demonstrate that DDX3X acts as a binding partner for Casein Kinase I Isoform Epsilon (CSNK1E) within the Wnt signaling pathway, implicating it in Wnt pathway regulation (Cruciat et al., 2013). However, whether *ddx3xa* exerts a conserved regulatory function in zebrafish remained unknown.

**FIGURE 8:**
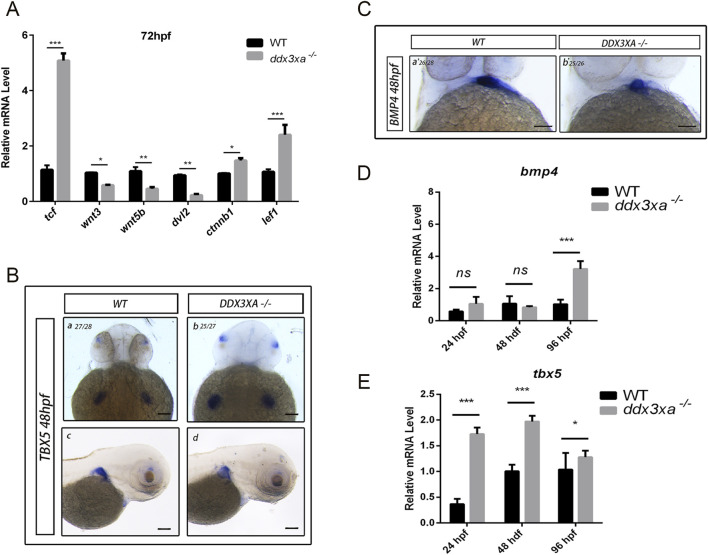
Wnt signaling disruption in *ddx3xa* ^−/−^ zebrafish. **(A)** Expression of key Wnt signaling components in *ddx3xa* ^−/−^ larvae at 72 hpf by qRT-PCR (n = 3; *p < 0.05, **p < 0.01, ***p < 0.001 vs. WT). **(B)** Whole-mount *in situ* hybridization (WISH) with *tbx5* probe at 48 hpf. Numbers indicate identically stained embryos/total processed. Scale bar: 250 μm (applies to B–C). (a) WT dorsal view; (b) *ddx3xa* ^−/−^ dorsal view; **(C)** WT lateral view; **(D)** *ddx3xa* ^−/−^ lateral view. **(C)** WISH with *bmp4* probe at 48 hpf. (a) WT ventral view; (b) *ddx3xa* ^−/−^ ventral view. **(D)** *bmp4* expression levels in *ddx3xa* ^−/−^ embryos at 24, 48, and 96 hpf by qRT-PCR (n = 3; ***p < 0.001 vs. WT; ns: nonsignificant). **(E)** *tbx5* expression levels in *ddx3xa* ^−/−^ embryos at 24, 48, and 96 hpf by qRT-PCR (n = 3; *p < 0.05, ***p < 0.001 vs. WT).

To directly assess the role of *ddx3xa* in the zebrafish Wnt pathway, we quantified the expression of key pathway components in mutants. qRT-PCR analysis showed that at 72 hpf, transcript levels of the downstream effector *tcf/lef1* and the core component *ctnnb1* (*β-catenin*) were significantly upregulated, whereas upstream components (*wnt3*, *wnt5b*, and *dvl2*) were significantly downregulated (Figure 8A). This provides the first evidence that *ddx3xa* mediates Wnt signal transduction through regulators like tcf/lef1, and its loss creates an imbalanced state characterized by aberrant downstream activation coupled with upstream suppression. We propose that this dysregulation particularly abnormal Tcf/Lef1 activity serves as a core mechanism underlying the misregulation of critical cardiac genes (*bmp4*, *tbx5*), ultimately driving cardiac defects.

Building on these findings, we investigated whether *ddx3xa* modulates cardiac development via the Wnt pathway. Whole-mount *in situ* hybridization (WISH) analysis of Wnt-associated cardiac genes *bmp4* and *tbx5* revealed spatial expression abnormalities: *tbx5* exhibited intensified dorsal labeling accompanied by expanded expression domains within the cardiac field (Figure 8B), while *bmp4* displayed pronounced atrial-specific expression expansion at 48 hpf (Figure 8C). These spatial anomalies provide direct support for the proposed mechanism.

To resolve developmental dynamics, we analyzed the temporal expression of these genes. In *ddx3xa* ^−/−^ mutants, *bmp4* was specifically upregulated at 96 hpf (Figure 8D), whereas *tbx5* showed sustained upregulation at all stages examined (24, 48, and 96 hpf; Figure 8E). Integrated spatiotemporal analysis demonstrates that *ddx3xa* deficiency not only disrupts the spatial patterning of Wnt-dependent cardiac genes but, more critically, disrupts their temporally regulated expression program, leading to dysregulated coordination of key factors essential for cardiac morphogenesis.

### 2.6 Rescue experiments confirm *ddx3xa* modulates cardiac development through wnt signaling

To further confirm that *ddx3xa* regulates cardiac development through the Wnt signaling pathway, we performed rescue experiments using IWR-1, a specific inhibitor of β-catenin/TCF interactions in the canonical Wnt pathway. Wild-type (WT), *ddx3xa* ^−/−^, *myl7:EGFP* transgenic line, and *ddx3xa* ^−/−^; *myl7:EGFP* double-homozygous embryos were treated with IWR-1, and cardiac phenotypes were assessed at 72 hpf.

**FIGURE 9:**
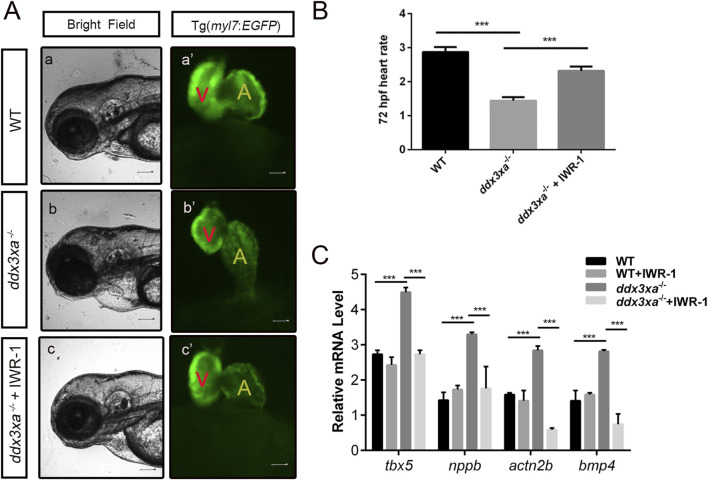
Pharmacological rescue of cardiac defects by Wnt inhibition. **(A)** Cardiac morphology at 72 hpf. Brightfield **(A–C)** and corresponding *myl7:EGFP* fluorescence (a'-c') views. (a,a') Wild-type (WT); (b,b') *ddx3xa* ^−/−^; *myl7:EGFP* homozygous mutant; (c,c') *ddx3xa* ^−/−^; *myl7:EGFP* mutant treated with IWR-1. Scale bar: 250 μm. **(B)** Heart rate quantification: *ddx3xa* ^−/−^ versus IWR-1-treated *ddx3xa* ^−/−^ mutants (n = 3; ***P < 0.001). **(C)** qRT-PCR analysis of Wnt-dependent cardiac genes (*tbx5*, *nppb*, *actn2b*, *bmp4*) in WT, WT + IWR-1, *ddx3xa* ^−/−^, and *ddx3xa* ^−/−^ +IWR-1 embryos at 72 hpf (n = 3; ***P < 0.001).

Untreated *ddx3xa* ^−/−^ embryos exhibited severe cardiac abnormalities, including pronounced pericardial edema, ventricular reduction with atrophy, atrial dilation, and overall cardiac elongation (Figure 9A; Supplementary Table S3). Notably, IWR-1 treatment significantly ameliorated cardiac malformations in *ddx3xa* ^−/−^ embryos at 72 hpf: pericardial edema was reduced, ventricular size was partially restored, atrial dilation was alleviated, and heart tube morphology appeared more compact and organized (Figure 9A; Supplementary Table S3). This phenotypic rescue was consistently observed in both bright-field and fluorescence views (*myl7:EGFP*-labeled cardiac tissue), confirming that Wnt pathway inhibition improves structural defects caused by *ddx3xa* deficiency.

Cardiac functional rescue was further validated by assessing heart rate. At 72 hpf, untreated *ddx3xa* ^−/−^ embryos showed significantly reduced cardiac contraction frequency (beats per 10 s) compared to WT. IWR-1 treatment markedly increased heart rate in *ddx3xa* ^−/−^ embryos, partially restoring cardiac function (Figure 9B).

To investigate the molecular basis of rescue, we analyzed expression of Wnt-downstream cardiac regulatory genes (*tbx5*, *nppb*, *actn2b*, *bmp4*) via qRT-PCR in four groups at 72 hpf: WT, WT + IWR-1, *ddx3xa* ^−/−^, and *ddx3xa* ^−/−^+IWR-1. Results demonstrated that IWR-1 treatment significantly downregulated *tbx5*, *nppb*, *actn2b*, and *bmp4* expression compared to untreated *ddx3xa* ^−/−^ embryos (Figure 9C). These molecular changes align with observed morphological improvements, confirming that IWR-1-mediated Wnt inhibition restores dysregulated expression of key cardiac developmental genes caused by *ddx3xa* knockout.

Collectively, these results demonstrate that pharmacological inhibition of Wnt signaling by IWR-1 not only rescues structural abnormalities (pericardial edema, ventricular atrophy, atrial dilation) but also improves cardiac function and normalizes critical cardiac regulatory gene expression in *ddx3xa* ^−/−^ zebrafish. This provides direct evidence that *ddx3xa* governs early cardiac development and function through modulation of the Wnt signaling pathway.

## 3 Discussion

This study provides the first systematic *in vivo* evidence establishing a direct causal link between DDX3X deficiency and cardiac developmental defects. Utilizing a CRISPR/Cas9-generated *ddx3xa* homozygous knockout zebrafish model (Sun et al., 2020), we recapitulated potential cardiac comorbidities observed in DDX3X syndrome patients. Mutant embryos exhibited a high malformation rate (25.26%) at 48–72 h post-fertilization (hpf), characterized by pericardial edema and defective cardiac looping (Figure 5), phenotypes highly concordant with clinically reported cardiac structural abnormalities (von Mueffling et al., 2024). Notably, coupled with early-onset developmental delay (Supplementary Figure S1), underscoring the fundamental role of DDX3X in embryonic development and organogenesis. The molecular fidelity of our model is supported by the high amino acid sequence homology (>75%) between zebrafish Ddx3xa and human DDX3X, particularly within the conserved RNA-binding domains (Figure 1). This model addresses a critical gap in DDX3X syndrome research, and its highly reproducible cardiac phenotypes -including atrial hypoplasia, ventricular dysmorphology, and impaired contractile function–provide an invaluable *in vivo* platform for dissecting the pathological mechanisms underlying *DDX3X* mutation-associated cardiac disorders in humans. Considering the clinical profile of DDX3X syndrome, our findings further suggest that cardiac abnormalities may represent an under-recognized comorbid phenotype warranting increased clinical attention.

**FIGURE 10:**
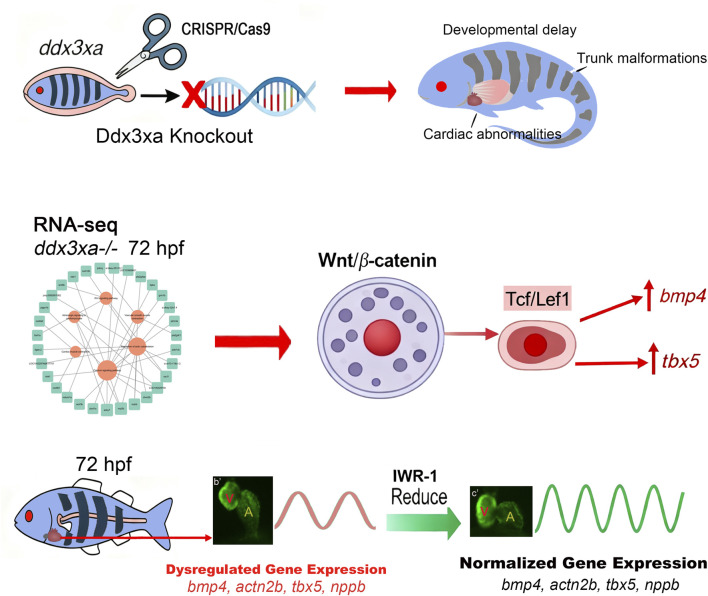
*ddx3xa* Governs Cardiac Morphogenesis Through Wnt/β-Catenin Signaling. Schematic diagram illustrating the mechanistic role of *ddx3xa* in zebrafish cardiac development. CRISPR/Cas9-mediated *ddx3xa* knockout results in developmental defects including developmental delay, trunk malformations, and cardiac abnormalities characterized by pericardial edema and impaired looping. Transcriptomic profiling of *ddx3xa* ^−/−^ mutants at 72 hpf identifies dysregulation of key cardiac developmental genes *(bmp4, actn2b, tbx5, nppb*) and reveals hyperactivation of the Wnt/β-catenin pathway. Mechanistically, *ddx3xa* deficiency upregulates the transcription factor Tcf/Lef1, leading to aberrant expression of its downstream targets *bmp4* and *tbx5*. Pharmacological inhibition of Wnt signaling with IWR-1 normalizes the expression of these cardiac genes and partially rescues the morphological defects. This work establishes *ddx3xa* as a critical regulator of cardiac development through modulation of the Wnt/β-catenin pathway, providing novel mechanistic insights into the cardiac comorbidities of DDX3X syndrome and highlighting the therapeutic potential of Wnt pathway modulation.

Based on our integrated findings, we propose a mechanistic model wherein *ddx3xa* governs cardiac morphogenesis through modulation of the Wnt/β-catenin signaling pathway (Figure 10).

Cardiac development relies on precise spatiotemporal regulation by intricate signaling networks (Liu and Foley, 2011). Our research findings approach identified the Wnt/β-catenin pathway as the central hub mediating the teratogenic effects of *ddx3xa* deficiency. Transcriptome sequencing revealed significant enrichment of the Wnt pathway in 72 hpf *ddx3xa* ^−/−^ embryos, concurrently dysregulated alongside actin cytoskeleton organization, calcium signaling, and cardiac muscle contraction pathways (Figure 6D). Mechanistically, *ddx3xa* knockout induced aberrant downregulation of the key Wnt transcriptional mediator Tcf/Lef1, while expression of upstream ligands (*wnt3*, *wnt5b*), cytoplasmic transducer (*dvl2*), and the effector *β-catenin* (*ctnnb1*) was paradoxically upregulated (Figure 8A). This “downstream activation-upstream suppression” paradox suggests that *ddx3xa* may regulate Tcf/Lef1 through non-canonical mechanisms. Crucially, severe spatiotemporal dysregulation was observed for Wnt-dependent cardiac target genes: *bmp4* showed abnormally high expression at 96 hpf concomitant with atrial enlargement (Figures 8C,D), while *tbx5* exhibited sustained upregulation and aberrant dorsal expansion within the heart field across multiple developmental stages (Figures 8B,E). Given that BMP4 drives cardiac chamber septation and TBX5 regulates atrioventricular differentiation, their dysregulated expression directly explains the observed atrial hypoplasia and looping failure in mutants (Figure 5). We propose that *ddx3xa*, potentially via its RNA helicase activity, could modulate the mRNA stability or translation efficiency of these target genes, or influence phase separation of Wnt pathway components (analogous to *ddx3xb*’s role in maternal-to-zygotic transition) (Shen et al., 2022; Shi et al., 2022), offering novel perspectives on how DDX3X fine-tunes the cardiac developmental gene network.

Cardiac contractile dysfunction, manifesting as bradycardia, was another prominent phenotype in *ddx3xa* mutants. Our study delineated a molecular-to-physiological cascade linking this defect to Wnt signaling dysregulation. qRT-PCR confirmed significant downregulation of key genes regulating myocardial contraction (*myh7*, *nppa*, *plcd1a*, *rac1*) and calcium signaling (*itpka*, *plcd1a*) in mutants (Figures 5T, 7A). These genes are directly involved in sarcoplasmic reticulum calcium cycling and myofilament sliding–for instance, PLCD1a hydrolyzes PIP2 to regulate calcium release, while RAC1 influences actin polymerization (Leung et al., 2014; Vachel et al., 2015). Their impaired expression resulted in significantly reduced heart rates (Figures 5Q–S), reflecting diminished contractile force. Intriguingly, KEGG analysis indicated co-enrichment of “Cardiac muscle contraction” and “Wnt signaling” pathways (Figure 6D), a connection substantiated by a pathway-gene network that integrated these enriched pathways and demonstrated strong interactions between Wnt downstream factors and contractile genes (Figure 7B). Experimentally, we demonstrated that aberrant Tcf/Lef1 upregulation suppresses the expression of *nkx2.5* and myosin genes (*myh7*, *myl7*) (Figures 5O,P,T), directly disrupting contractile unit assembly. Furthermore, persistent Wnt/β-catenin activation is known to inhibit cardiomyocyte differentiation and promote fibrosis (Garcia-Gras et al., 2006). Our finding of similar phenotypes in *ddx3xa* mutants suggests that DDX3X deficiency, via chronic Wnt hyperactivation, may drive cardiomyocytes towards pathological remodeling, providing mechanistic insights into potential arrhythmogenic risks in DDX3X syndrome patients.

The most pivotal breakthrough of this study lies in the phenotypic rescue achieved through targeted Wnt pathway intervention, providing direct evidence for causality. Treatment of 72 hpf mutant embryos with the specific Wnt/β-catenin inhibitor IWR-1 resulted in: (1) Morphological Rescue: Significant reduction in pericardial edema, marked improvement in ventricular hypoplasia and atrial enlargement, and partial restoration of cardiac looping (Figure 9A). (2) Functional Recovery: Substantial increase in heart contraction frequency (Figures 9B,C), indicating enhanced pumping capacity. (3) Molecular Correction: Effective suppression of aberrantly high expression of Wnt-dependent cardiac genes (*tbx5*, *nppb*, *actn2b*, *bmp4*) (Figure 9D). This multi-level rescue unequivocally confirms that the cardiac defects in *ddx3xa* mutants are fundamentally driven by hyperactivated Wnt/β-catenin signaling. Notably, IWR-1 acts upstream of Tcf/Lef1 by stabilizing the AXIN complex to promote β-catenin degradation (Lu et al., 2009). Its ability to downregulate Tcf/Lef1 target genes (*bmp4*, *tbx5*) demonstrates that the aberrant Tcf/Lef1 activation caused by *ddx3xa* deficiency is reversible via upstream intervention. This offers proof-of-concept for treating DDX3X syndrome-associated cardiac disease-small-molecule Wnt inhibitors (IWR-1) represent potential therapeutic strategies. The partial nature of the rescue (incomplete normalization of cardiac morphology) suggests that *ddx3xa* may also co-regulate cardiac development through additional pathways (calcium signaling, adrenergic signaling; Figure 7), necessitating combined targeted interventions for optimal efficacy.

This study is the first to integrate DDX3X deficiency, Wnt signaling dysregulation, and cardiac maldevelopment within a unified mechanistic framework, opening new dimensions for DDX3X syndrome research. The high-throughput capabilities of the zebrafish model provide a powerful platform for accelerating mechanistic dissection and therapeutic development. However, several key questions warrant further investigation: (1) Precise Molecular Interface of *ddx3xa*-Wnt Regulation: Does DDX3X directly bind Wnt pathway components (CSNK2A1 or β-catenin mRNA)? How does its helicase activity influence Tcf/Lef1 transcriptional complex assembly? (2) Cardiac-Specific Mechanisms: Why does *ddx3xa* deficiency selectively impact the heart despite expression in other organs? Tissue-specific knockout or single-cell sequencing is needed to elucidate cell-autonomous effects in cardiomyocytes. (3) Sex-Specific Considerations: Given the female predominance of DDX3X syndrome and the autosomal location of zebrafish *ddx3xa*, the compensatory effects of the DDX3Y homolog and the influence of sex hormone microenvironments require exploration. (4) Functional Validation of Clinical Mutations: Introducing patient-derived cardiac-associated *DDX3X* mutations into the zebrafish model is crucial to assess their specific impact on Wnt signaling and cardiac phenotypes.

In conclusion, this work not only establishes the central role of the *ddx3xa*-Wnt axis in cardiac development but also builds a bridge connecting fundamental research to clinical translation. Future integration of patient-derived induced pluripotent stem cell (iPSC)-cardiomyocyte models with *in vivo* zebrafish screening holds significant promise for developing precision therapeutic strategies targeting cardiac comorbidities in DDX3X syndrome.

## 4 Materials and methods

### 4.1 Zebrafish husbandry

Wild-type AB strain zebrafish (*Danio rerio*) were maintained at 28.5 °C ± 0.5 °C under a 15-h light/9-h dark photoperiod. Embryos were reared in E3 embryo medium (pH 7.0; conductivity ≈600 μS/cm) until 7 days post-fertilization (dpf), fed Paramecium from 1-7 dpf and Artemia nauplii thereafter, then transferred to recirculating aquaculture systems (Aisheng Technology).

### 4.2 Reagents and instrumentation

Key reagents included: FastPure RNA Purification Kit (Vazyme), dNTP Mix (Takara), anti-DDX3X antibody (Genecopoeia), Cas9 protein (Thermo Fisher), restriction enzymes (Promega), and T7 Transcription Kit (Promega). Major instruments comprised: PCR Thermal Cycler (Eppendorf), Microinjection System (Harvard Apparatus PLI-100A), Confocal Microscope (Leica), and qPCR System (Eppendorf) (Complete lists: Supplementary Tables S4,S5).

### 4.3 Primer design

sgRNA target sites for *ddx3xa* knockout were designed via ZIFIT (avoiding UTRs; NGG PAM), while qRT-PCR primers were designed using Primer Premier 5.0 (Supplementary Table S6).

### 4.4 Generation of *ddx3xa* mutants

sgRNAs were synthesized by PCR-amplifying T7 promoter-containing templates (Supplementary Table S3), followed by *in vitro* transcription (T7 Kit) and purification (FastPure RNA Kit). For microinjection, 500 pg sgRNA and 300 pg Cas9 protein were co-injected into one-cell embryos, with injected embryos incubated in E3 medium at 28 °C.

### 4.5 Genotyping

Genomic DNA was extracted from embryos/fin clips using alkaline lysis (50 mM NaOH, 95 °C, 10 min). Target regions were amplified by PCR (primers *ddx3xa*-F/R) and mutations verified via Sanger sequencing.

### 4.6 Transcriptome profiling via RNA-Seq

Zebrafish embryos at 72 hpf were batched into biological replicates (50 embryos per tube) and flash-frozen at −80 °C. RNA sequencing was performed commercially by Personalbio GeneCloud (Shanghai, China), with subsequent bioinformatic analyses executed on their proprietary platform (https://www.genescloud.cn/home). Pathway-gene target networks were visualized using Cytoscape software.

### 4.7 Imaging and quantitative analysis

Cardiac morphology in *myl7:EGFP* transgenics was documented by fluorescence stereomicroscopy (Leica M205FA). Whole-mount *in situ* hybridization specimens were imaged using a Leica digital system (v3.2.0), while larval phenotypes (24–48 hpf) were captured with a motorized Axiocam (Zeiss) and processed using AxioVision 3.0.6 and Adobe Photoshop. Cardiac function was assessed by recording ventricular contractions at 130 fps for 10-s intervals via high-speed EM-CCD camera (×48 magnification), with semi-automated analysis generating quantitative parameters including heart rate, visualized through M-mode imaging.

### 4.8 Quantitative RT-PCR (qRT-PCR)

Total RNA was extracted (FastPure Kit), reverse-transcribed (Hifair III SuperMix), and amplified (SYBR Green Master Mix; Eppendorf). Using β-actin as reference gene, data were analyzed in GraphPad Prism 6.0 via Student’s t-test (P < 0.05 significance threshold).

### 4.9 Whole-mount in situ hybridization (WISH)

Digoxigenin-labeled antisense probes (500–1000 bp spanning *ddx3xa* 3′-UTR) were transcribed from PCR templates. Embryos were fixed in 4% PFA, dehydrated/rehydrated, treated with proteinase K (stage-optimized concentration/duration), pre-hybridized (65 °C, 4–6 h), and hybridized overnight (65 °C). Post-hybridization washes included sequential treatments with 50% formamide/2× SSCT, 2× SSCT, and 0.2× SSCT. After blocking, embryos were incubated with anti-DIG-AP antibody (1:3,000) and developed with NBT/BCIP, with images acquired by stereomicroscopy.

### 4.10 Pharmacological rescue

Adult zebrafish were sex-separated (1♀:1♂ or 1♀:2♂ ratio) in breeding tanks with transparent dividers, dark-adapted for ≥6 h, then paired under light conditions. At 6 hpf, embryos were exposed to control solution (0.05% DMSO in E3) or experimental treatment (10 nM IWR-1 in E3) in 6-well plates (20 embryos/well). Malformation and mortality rates were recorded at 24, 48, and 72 hpf, with heart rate and ejection fraction analyzed at 72 hpf via high-speed videomicroscopy.

### 4.11 Statistical analysis

All experiments included ≥3 biological replicates, with statistical significance determined by Student’s t-test (P < 0.05).

## Data Availability

The original contributions presented in the study are included in the article/Supplementary Material, further inquiries can be directed to the corresponding authors.
