# Supplementary material for: Ddx3xa mutations drive cardiac defects in a zebrafish model via dysregulation of wnt/β-catenin signaling
Source: Front Mol Biosci. 2025 Nov 27;12:1689202. doi: 10.3389/fmolb.2025.1689202 (PMC12696674; doi:10.3389/fmolb.2025.1689202)
Supplement: Supplementary file 1 [file DataSheet1.docx]

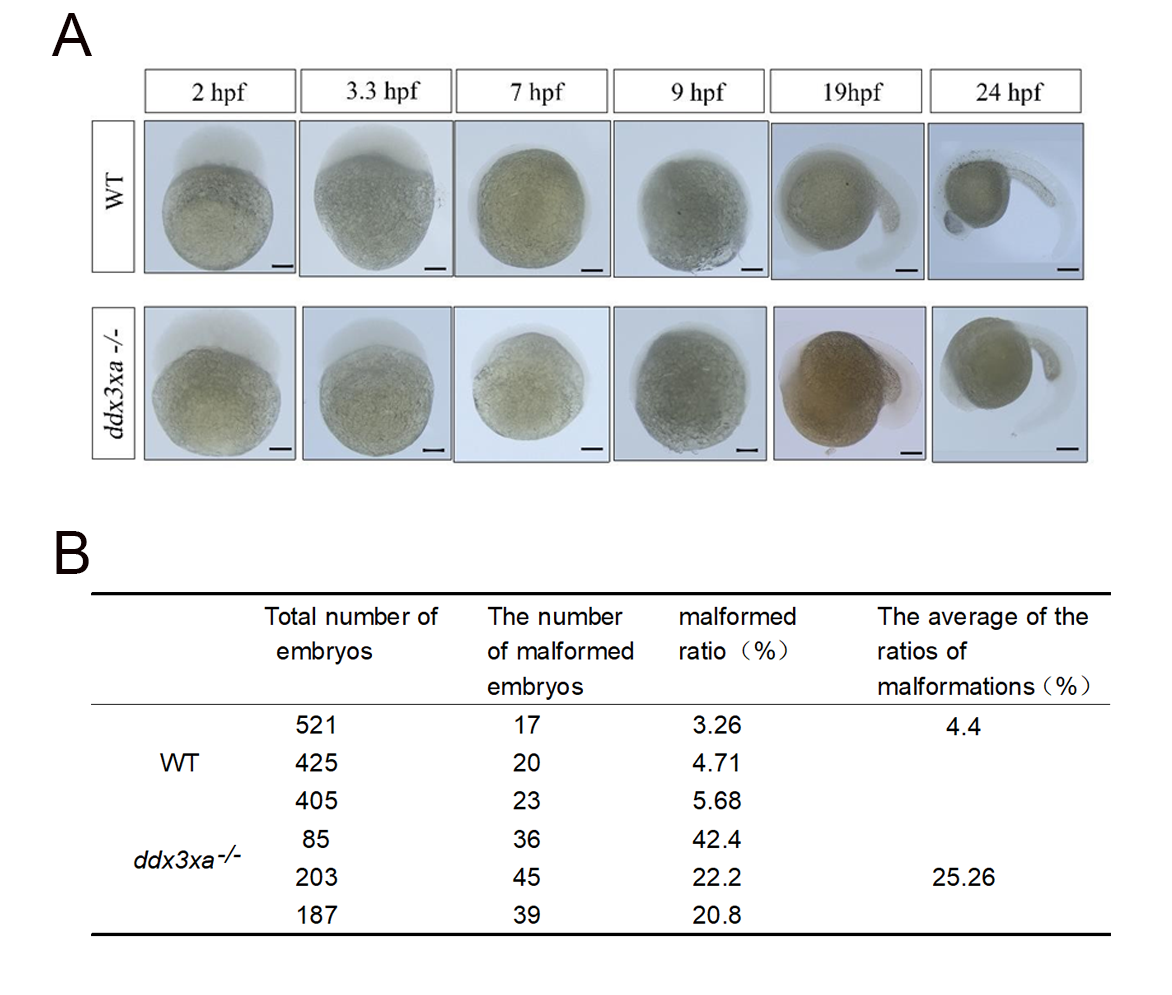


**Figure S1. Phenotypic analysis of *ddx3xa^−/−^* zebrafish embryos.**(A) Representative developmental phenotypes of wild-type (WT) and *ddx3xa^−/−^* embryos at 24 hpf. Scale bar: 300 μm.
(B) Quantification of malformation rates in *ddx3xa^−/−^*  mutants versus WT controls.


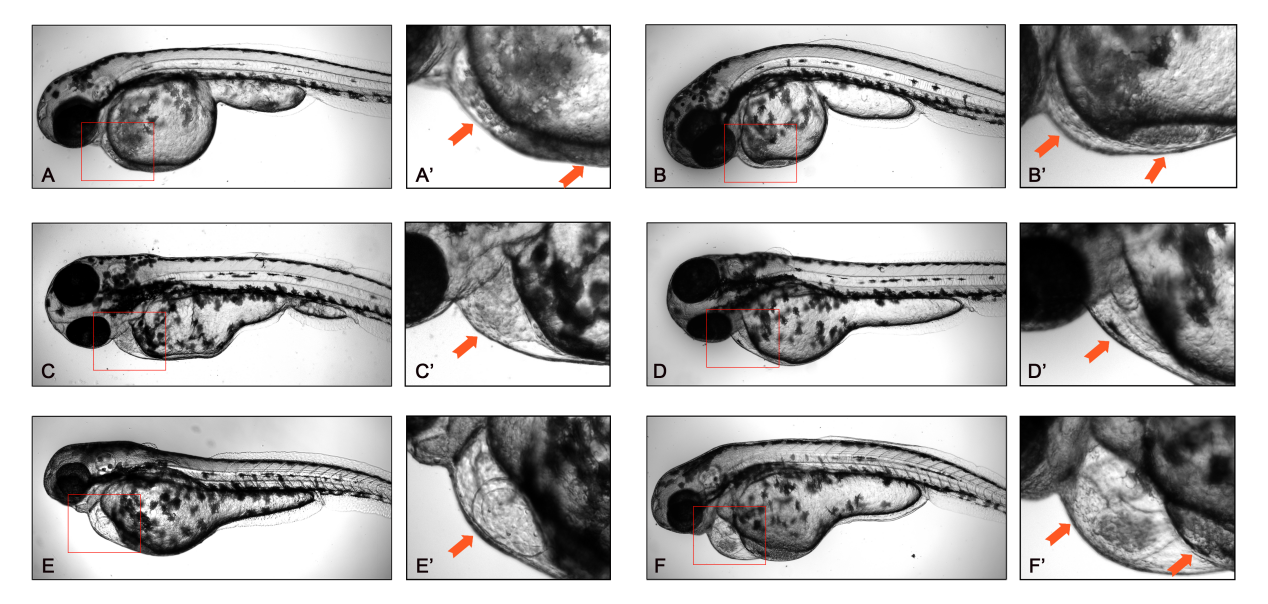
**Figure S2. *ddx3xa*^−/−^ homozygous mutant zebrafish exhibit significant cardiac developmental phenotypes at 48 hpf, including pericardial edema, failure of cardiac linearization, and impairment of cardiac looping formation.** Representative images show cardiac malformations of varying severity: (A, A', B, B') mild malformations with slight hemorrhage in the pericardial cavity; (C, C', D, D') moderate malformations with exacerbated pericardial edema and significant cardiac morphological abnormalities; (E, E', F, F') severe malformations characterized by severe pericardial edema and distortion of cardiac structure. Red arrows indicate the specific sites of cardiac malformations in each sample.

**Table S1. Quantitative analysis of cardiac malformations in *ddx3xa*^-/-^ zebrafish at 48 hpf**

|  | Total number of embryos | Pericardial edema number^[[1]](#footnote-1)^(percentage) | Cardiac linearization number^[[2]](#footnote-2)^(percentage) | Impaired looping process number^[[3]](#footnote-3)^(percentage) | The average of the ratios of malformations(%) |
| --- | --- | --- | --- | --- | --- |
| wt | 521 | 16(3.07%) | 0 | 1(0.19) | 4.4 |
|  | 425 | 17(4.00%) | 1(0.23%) | 2(0.47%) |  |
|  | 405 | 16(3.95%) | 2(0.49%) | 5(1.23%) |  |
| *ddx3xa^−/−^* | 85 | 14(16.47%) | 10(11.76%) | 12(14.11%) | 25.26 |
|  | 203 | 21(10.34%) | 9(4.43%) | 15(7.38%) |  |
|  | 187 | 19(10.16%) | 9(4.81%) | 11(5.88%) |  |


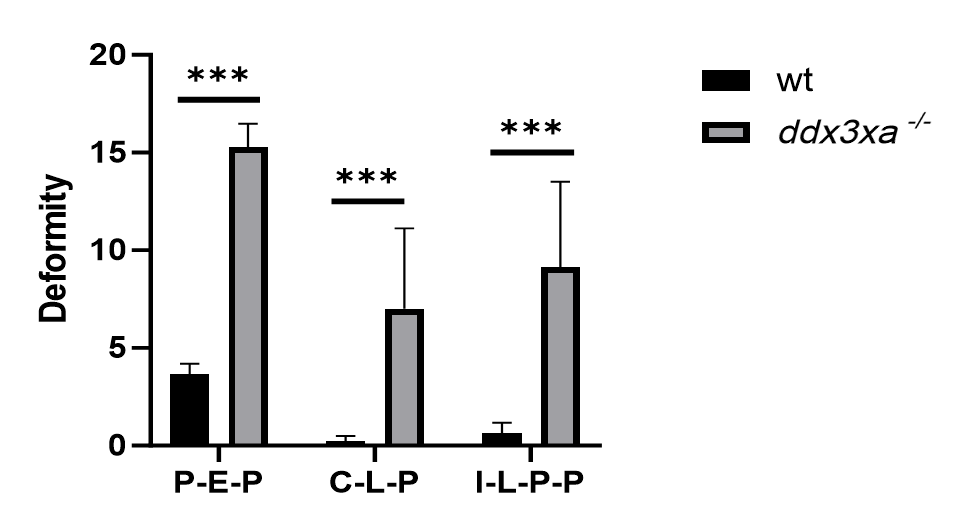


**Figure S3.** Significantly increased incidence of cardiac malformations (pericardial edema percentage(P-E-P), cardiac linearization percentage(C-L-P), and impaired looping process percentage(I-L-P-P)) in *ddx3xa^-/-^* zebrafish at 48 hpf (***P<0.001)。

**Table S2. The list of up- and down-regulated candidate genes（In the supplement）**

**Table S3. Quantitative analysis of cardiac malformations in WT, *ddx3xa*^−/−^ and *ddx3xa*^−/−^ + IWR-1 at 72 hpf**

|  | Total number of embryos | Pericardial edema number^[[4]](#footnote-4)^(percentage) | Cardiac linearization number^[[5]](#footnote-5)^(percentage) | Impaired looping process number^[[6]](#footnote-6)^(percentage) | The average of the ratios of malformations(%) |
| --- | --- | --- | --- | --- | --- |
| WT | 384 | 16(4.17%) | 1(0.26%) | 2(0.52%) | 4.5 |
|  | 475 | 15(3.16%) | 1(0.21%) | 4(0.84%) |  |
|  | 451 | 19(4.21%) | 0 | 1(0.22%) |  |
| *ddx3xa^−/−^* | 261 | 38(14.56%) | 17(6.51%) | 14(5.36%) | 27.08 |
|  | 147 | 21(14.28%) | 9(6.12%) | 11(7.48%) |  |
|  | 334 | 53(15.87%) | 17(5.09%) | 21(6.29%) |  |
| *ddx3xa^−/−^+*IWR-1 | 211 | 9(4.27%) | 1(0.47%) | 3(1.42%) | 7.23 |
|  | 168 | 13(7.74%) | 0 | 1(0.60%) |  |
|  | 257 | 12(4.67%) | 2(0.78%) | 5(1.95%) |  |

**Table S4. Reagents for Molecular Biology**

| **Reagent** | **Company** |
| --- | --- |
| Citric acid | ComWin Biotech |
| dNTPs | Takara |
| DDX3X Antibody | Jiekewei Biotechnology |
| DNA Marker | Yeasen Biotechnology |
| 2×Taq Master Mix（Dye） | ComWin Biotech |
| Phanta ® Max Super-Fidelity DNA Polymerase | Novizan Scientific |
| Ribonucleic acid | SIGMA |
| Super Gel Red Nucleic Acid Stain | Sangon Biotech |
| Citric acid | Sinopharm |
| Agar A | SIGMA |
| Formamide | SIGMA |
| RNA-free absolute ethanol, RNA-free methanol | Sinopharm |
| Primer synthesis and sample sequencing | Ucome Biotech、Tsinke Biotech |
| First-strand cDNA Synthesis Mix | Lanbolide |
| Reverse transcription kit | Fermentas |
| TrueCut™ Cas9 Protein v2 | Thermo Fisher |
| Restriction endonuclease | Promega |
| pMD18-T | YEASEN |
| Quick Agarose Gel DNA Recovery Kit | ComWin Biotech |
| T7 Transcription Kit | Promega |
| RNeasy Mini kit | Qiagene |
| Yeast extract | BBI |
| Trytone | BBI |

**Table S5. Major Laboratory Equipment**

| **Instrument names** | **Company** |
| --- | --- |
| PCR machine, pipette, spectrophotometer | Eppendorf |
| Sterile filter | Millipore |
| Centrifuge (including 4°C and large-capacity models) | Fresco |
| Gel imaging analysis system | Tanon |
| DYY-6C vertical slab electrophoresis tank | Liuyi Instrument Factory |
| Small animal ultrasound system | Fuji Medical Technology Service Co., Ltd. |
| Constant temperature shaking incubator (HZQ-A) | Jiangsu Taicang Instrument and Equipment Company |
| -80°C upright refrigerator | Thermo |
| Ice maker | Ningbo Xinzhi Company |
| Phenotype analysis multi-zoom microscope | Olympus Corporation |
| Stereomicroscope | Leica Microsystems |
| Micropipette puller | narishige |
| Microinjection system (PLI-100A) | Harvard Apparatus |
| Inverted fluorescence stereomicroscope | Leica Microsystems |
| Precision analytical balance | Sunny Hengping Science & Technology Co., Ltd. |

**Table S6. Primer Sequences**

| primer | Sequence (5'→3') |
| --- | --- |
| *ddx3xa*-Guide RNA-F1 | tgTAATACGACTCACTATAtgggatggtagtcgtaccaaGTTTTAGAGCTAGAAATAGC |
| *ddx3xa*-Guide RNA-R1 | tgTAATACGACTCACTATAcctcttcgcaatgaccgaggGTTTTAGAGCTAGAAATAGC |
| *ddx3xa*-sgRNA-R | AAGCACCGACTCGGTGCCACT |
| *ddx3xa*-F | GGTTCCTTACTCCGCTATTC |
| *ddx3xa*-R | ACAAAGATGGAGGCTGGA |
| *ddx3xa*-qRT-PCR-F | TGGGATGGTAGTCGTACCAATGG |
| *ddx3xa*-qRT-PCR-R | GGCATCTCTGTTCACCACAGAG |
| *ddx3xa*-wish-F | CACCCAGCAGGAGAATGGAA |
| *ddx3xa*-wish-R | TAATACGACTCACTATAGGGAGAGAATCCTTGCCTGTGGC |
| q-z-*β-actin*-F | TCCAGCCTTCCTTCCTGGGTAT |
| q-z-*β-actin*-R | GTGGAAGGAGCAAGAGAGGTG |
| q-z-*nppa*-F | TCAGAGGGCAAGAAAACGCA |
| q-z-*nppa*-R | AGCACTGACTGTTTACCTCACAT |
| q-z-*myl7*-264-F | GCTCTGGGTGTCCATGTAGG |
| q-z-*myl7*-264-R | TATGCAGCCAAAAGCCTCCT |
| q-z-*gata4*-220-F | TGCTAGACACCCCAATTTAGAGTT |
| q-z-*gata4*-220-R | TGTGCAGGATAAACCCACCC |
| q-z-*nppb*-297-F | ACAAACGACGACATGGGTGT |
| q-z-*nppb*-297-R | GTTCTTCTTGGGACCTGAGC |
| q-z-*actn2b*-277-F | TCTCCGGTCGAGAGAGTCTG |
| q-z-*actn2b*-277-R | AGGCAGTGAACGTCTTCCTT |
| q-z-*bmp4*-121-F | GCAGTGCCTTCAAAGGTTGG |
| q-z-*bmp4*-121-R | GCGGGGAGATCCTTTTCCATT |
| q-z-*ntrk1*-115-F | ATTGACCCACCGACAGTGAC |
| q-z-*ntrk1*-115-R | CCAGATTCCTTGCTCCAGGG |
| q-*pla2g4aa*-112-F | CGATAGCGCAAGTCCCTCAC |
| q-*pla2g4aa*-112-R | TGTCTACCGTGAGGCCTTAAA |
| q-*pla2g4f.1*-106-F | CGCCTTTTCCATACTCAGGC |
| q-*pla2g4f.1*-106-R | CAGTATGGCTTGGCTTCTTTCTTA |
| q-z-*plcd1a*-298-F | ACGGGCTTTCGTGAAGGATT |
| q-z-*plcd1a*-298-R | TCCGCAAGTTCAAAGCTGGA |
| q-z*-ptger1b*-228-F | TGGAGCCCTCTGCTGATCTT |
| q-z-*ptger1b*-228-R | GAATGTGCTCCCTTTGCGTC |
| q-z-*ntsr1*-115-F | CACTCCGATGCTCTTCACGA |
| q-z-*ntsr1*-115-R | TGACAGGAAGGCATTCAACTGT |
| q-z-*itpka*-111-F | GCAGCAGAAGGCGGTTACTA |
| q-z-*itpka*-111-R | TTGCAGGTTTCCCCTCTCTTG |
| q-z-*p2rx3a*-252-F | AATTTGGTGTCGCTCGTGGT |
| q-z-*p2rx3a*-252-R | AGCATCCAAGCGTGTGAAGA |
| q-z-*adcy7*-289-F | ACCAACTCACAATCCAGACAGT |
| q-z-*adcy7*-289-R | GCCCAGCTCTATACGTTGCT |
| q-z-*rac1*-291-F | TAAGCCTGAGGAAAGGAGTGTG |
| q-z-*rac1*-291-R | ACACAGTGGGGACATAACCA |
| q-z-*chrm2b*-178-F | GCTTCAGATACCACTACAGGACT |
| q-z-*chrm2b*-178-R | AAGCTGATTGGTTCTGGCGA |
| q-z-*myh7l*-F | ACTACGCTGGCACAGTAGAC |
| q-z-*myh7l*-R | CCATCACCCGTTGCTGAGTC |
| q-z-*nkx2.5*-177-F | CGGATCCTCTCTCTTCAGCG |
| q-z-*nkx2.5*-177-R | TGACAACAGCCGATGTCTTTTT |
| q-z-*tbx20*-116-F | GCGAGGAGATGGCCAAGATT |
| q-z-*tbx20*-116-R | TGTCGGAAACATTCGTCTTCCA |
| q-z-*cxcl12b*-173-F | AGCCCAGAGACTGACGCAAA |
| q-z-*cxcl12b*-173-R | GGGTTGATGCAGACCTCTCT |

1. Defined as the accumulation of fluid in the pericardial cavity, but with normal cardiac looping (i.e., not meeting the criteria for the other two categories). This category exclusively includes embryos exhibiting edema alone. [↑](#footnote-ref-1)
2. Defined as a heart that remains completely or nearly completely as a linear tube, with no signs of looping. As the most severe phenotype, embryos meeting this criterion are classified into this category upon identification. [↑](#footnote-ref-2)
3. Defined as a heart that undergoes bending but fails to form the normal S-shaped structure (e.g., exhibiting insufficient looping angle or abnormal chamber alignment). This category includes only those embryos that do not meet the criteria for "cardiac linearization" but still display defective looping. [↑](#footnote-ref-3)
4. Defined as the accumulation of fluid in the pericardial cavity, but with normal cardiac looping (i.e., not meeting the criteria for the other two categories). This category exclusively includes embryos exhibiting edema alone. [↑](#footnote-ref-4)
5. Defined as a heart that remains completely or nearly completely as a linear tube, with no signs of looping. As the most severe phenotype, embryos meeting this criterion are classified into this category upon identification. [↑](#footnote-ref-5)
6. Defined as a heart that undergoes bending but fails to form the normal S-shaped structure (e.g., exhibiting insufficient looping angle or abnormal chamber alignment). This category includes only those embryos that do not meet the criteria for "cardiac linearization" but still display defective looping. [↑](#footnote-ref-6)
